# What policymakers want: policy briefs as tools to inform animal disease control strategies

**DOI:** 10.3389/fvets.2026.1813832

**Published:** 2026-05-21

**Authors:** Katherine Adam, Davide Pagnossin, Mayumi Fujiwara, Harriet Auty, Lisa Boden

**Affiliations:** 1 Royal (Dick) School of Veterinary Studies, University of Edinburgh, Edinburgh, United Kingdom; 2 School of Biodiversity, One Health and Veterinary Medicine, University of Glasgow, Glasgow, United Kingdom

**Keywords:** disease control, evidence-based decision-making, knowledge brokering, policy briefs, policymaking

## Abstract

**Introduction:**

Policy briefs are concise documents designed to communicate information to policymakers to inform the policymaking process. However, there is limited empirical evidence to guide scientists in writing effective policy briefs, and none to address the role of policy briefs in communicating animal health research findings specifically.

**Methods:**

In this qualitative study, we conducted semi-structured interviews with 14 decision-makers from Scottish Government and other United Kingdom administrations to gain insights into their experiences and perceptions of policy briefs relating to animal disease control. Two main areas were explored: the desired content and format of policy briefs, and the role of policy briefs in science-policy engagement.

**Results:**

The importance of achieving a balance between conflicting demands in content and formatting (e.g., including sufficient, accessible information, while excluding excessive detail) was identified as a key aspect of policy brief preparation. Policy briefs were identified as an important component of effective science-policy communication within the context of a trusting relationship between scientists and policymakers. The insights from decision-makers captured in this study have informed a set of recommendations for scientists developing policy briefs, particularly in the field of animal health.

## 1 Introduction

Collaboration between scientists and policymakers is becoming increasingly important for both parties. Policymakers recognise the need for scientific input to inform decision-making on health, social and environmental issues, as seen with the COVID-19 pandemic and the climate crisis (1, 2). Scientists, on the other hand, are motivated to increase the impact of their research and feel a responsibility to use their expertise to benefit society (3). Moreover, as trust in science has been increasingly questioned by the public in recent years, society now expects higher standards of collaboration between scientists and policymakers to achieve evidence-based solutions (4). A significant barrier to successful collaboration between science and policymaking lies in effective communication. Scientists often struggle to share their research in ways that are accessible to a non-academic audience, while policymakers may find it challenging to understand the limitations inherent in scientific research, the role of uncertainty in science, and extract key messages from often lengthy research reports (3).

Policy briefs (also sometimes referred to as policy briefings) are an important tool for communicating knowledge to policymakers within government, with the aim of informing policy decisions (5). Policy briefs are generally short, accessible summaries of information relevant to a specific policy decision or process. Due to their format and content, policy briefs have become one of the preferred ways to communicate science to policymakers (6). Although widely used, the role of policy briefs in knowledge brokering at the science-policy interface has received limited attention as a research subject, particularly in the context of animal health. Most of the current knowledge on effective communication between science and policy does not focus specifically on policy briefs (3). While guidelines for writing policy briefs are available in the literature, they are often based on studies of other communication tools (6). Policymakers’ feedback on the specific use of policy briefs to communicate scientific knowledge has previously been empirically investigated in other contexts (7, 8), but to the authors’ knowledge, policymakers’ preferences for using policy briefs to communicate knowledge in the veterinary field have not yet been explored.

**Figure 1:**
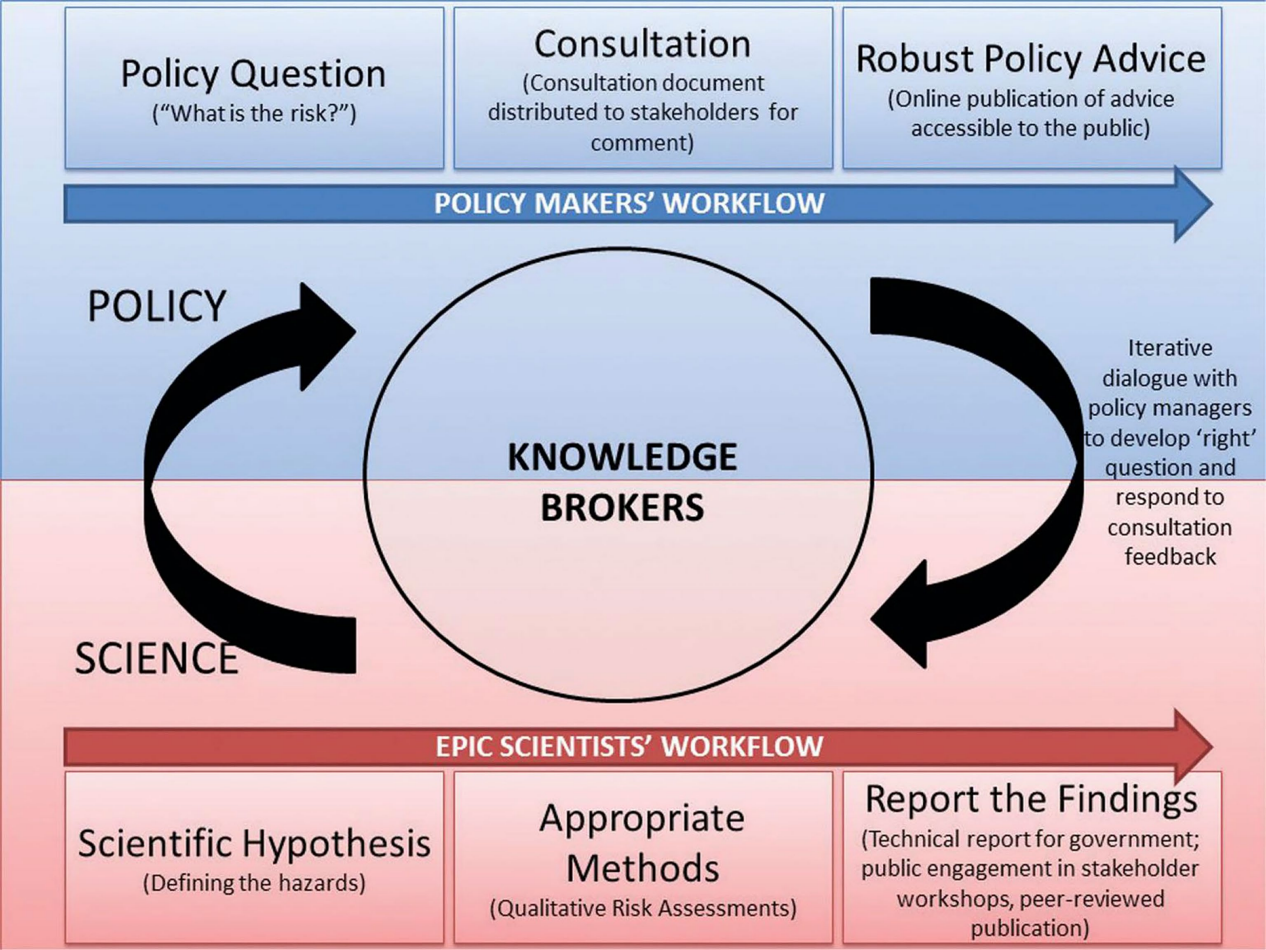
EPIC’s work at the science-policy interface [from (10), licensed under CC BY 4.0].

EPIC Scotland (the Scottish Government’s Centre of Expertise on Animal Disease Outbreaks) is an innovative model for the provision of risk-based evidence to policy (9, 10). The research consortium consists of epidemiologists, mathematicians, statisticians, economists, ecologists, veterinarians and social scientists from research institutions across Scotland, with additional administrative, project management and communications support. EPIC often communicates complex scientific information, derived from both quantitative and qualitative animal health research, to decision-makers within Scottish Government. Scientists working directly at the science-policy interface within EPIC aim to optimise the processes and practices for effective communication between scientists and policymakers to provide relevant evidence to inform policy decisions, as shown in Figure 1, (9). Policy briefs have been used extensively to share EPIC research outputs with Scottish Government policymakers for many years. However, beyond informal feedback mechanisms between EPIC scientists and policymakers, this is our first formal investigation of the efficacy of policy briefs in communicating scientific outputs.

This study aims to explore decision-makers’ experiences and perceptions of policy briefs in relation to decision-making on animal health and disease control. The research was conducted within a Scottish context and in relation to animal health policy, but the findings are likely to be of interest and relevance to scientists and stakeholders working in other fields and settings, and our research contributes to the growing body of literature on optimizing communication at the science-policy interface. In addition to exploring a previously understudied area of science-policy communication, the research findings were also used to develop practical recommendations to improve the use of policy briefs in the communication process between scientists and policymakers. These recommendations are presented at the end of this paper.

## 2 Materials and methods

This study is based on a series of semi-structured interviews with decision-makers involved in animal health policy within the United Kingdom. The study design and questionnaire were reviewed and approved by the Human (Research) Ethical Review Committee at the Royal (Dick) School of Veterinary Studies, the University of Edinburgh (reference number: HERC_747_21) and by the Scottish Government Rural and Environment Science and Analytical Services Division (RESAS). Informed, written consent was sought from all participants and recorded using an approved consent form.

### 2.1 Interview design

Due to the limited extant literature on this subject in relation to animal health, and as the research is situated in established working relationships, the interview schedule design and the topics addressed in the interviews were informed primarily by the authors’ extensive personal knowledge and experience of working at the science-policy interface in animal health and of the preparation of policy briefs (9, 10). Preliminary conversations with the policy audience for EPIC’s work, as well as informal feedback on previously supplied policy outputs from EPIC, also informed the study design and the topics covered in the semi-structured interviews.

**Figure 2:**
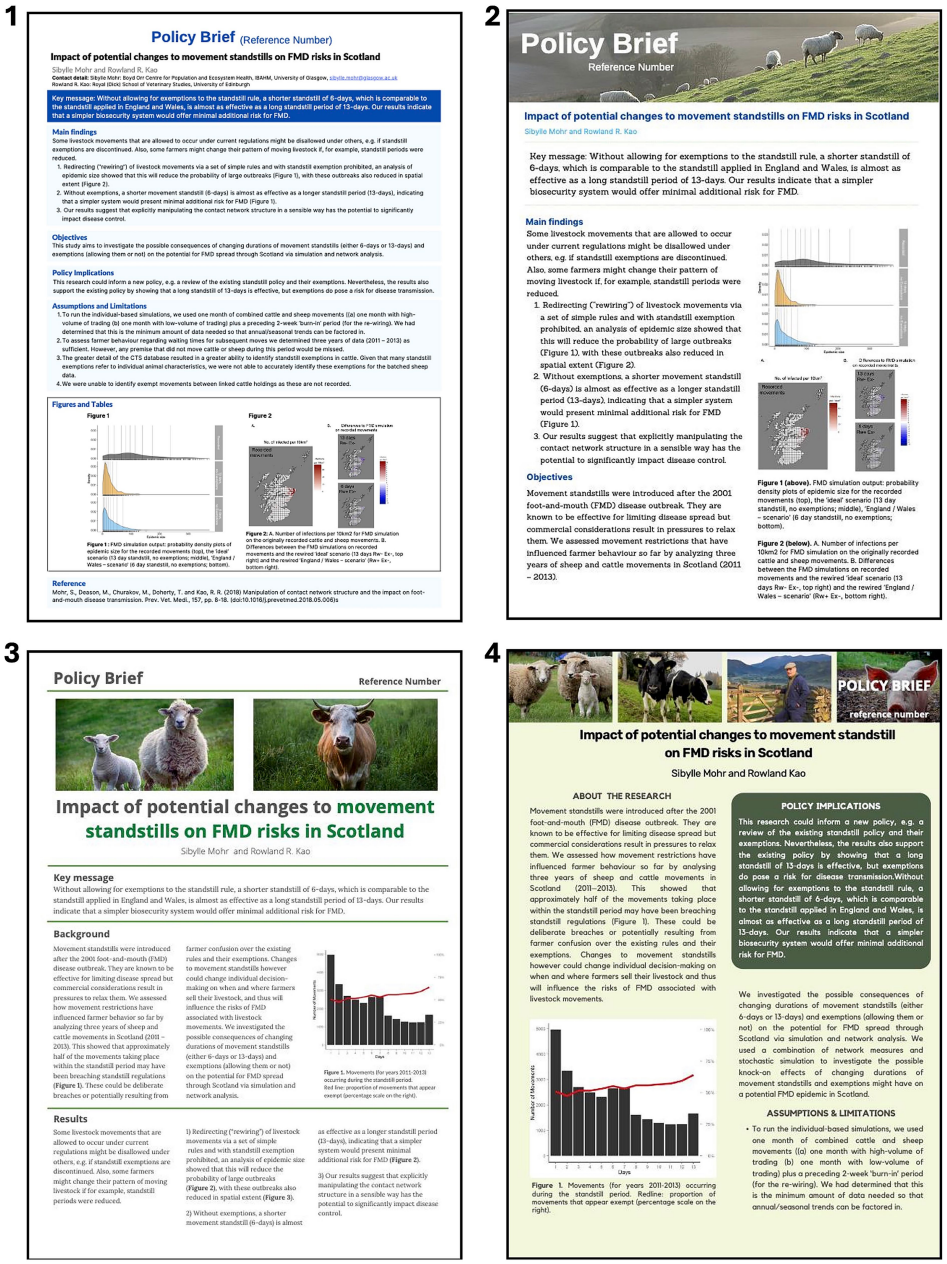
Policy brief templates presented to interviewees.

The interview design was based around four example policy briefs, all of which contained the same factual information, but presented in different ways in terms of the layout and design (Figure 2). The topic and content of all example templates related to research findings on the risk of foot and mouth disease (FMD) spread in Scotland in the event of an outbreak associated with potential changes to regulations on live animal movements. The decision to base the interviews on example templates was intended to maintain focus during the discussions and support detailed exploration of the reader’s experience of the same information in different formats. The simultaneous viewing of the examples during the interview was designed to ground both the interviewer and interviewee in a shared understanding of the interviewees’ personal preferences and specific feedback on the examples presented.

The structure of the interview schedule was designed as an “inverted funnel,” moving from highly specific discussion of the examples to more general exploration of policy briefs; this is a variation on the more common funneling technique of moving from the general to the specific in the course of a qualitative interview. Interview questions were piloted virtually using Microsoft Teams with two academic researchers with expertise in fields other than animal health to evaluate the effectiveness of the questions and to estimate the time of the interview. This led to the inclusion of two additional questions and minor text edits.

The full interview schedule (see Supplementary Materials) comprised four sections. Section 1 captured background information on respondents, including their organizational affiliation and role (questions 1.1 and 1.2), professional experience (questions 1.3 and 1.4), and areas of expertise (question 1.5). It also gathered information on their familiarity with EPIC and its outputs (questions 1.6 and 1.7), as well as their educational background (question 1.8), providing context for interpreting their responses.

Section 2 examined how respondents engage with and evaluate policy briefs, using a preferred template as a reference point (Figure 2). It explored the features that make a brief appealing (question 2.1) and the clarity and effectiveness of key messages (question 2.2), alongside the clarity and level of detail of core components, including background and objectives (question 2.3), the results and their presentation (question 2.4), and methodology (question 2.5). It further considered how assumptions and limitations are presented and used in decision-making (question 2.6), as well as perceptions of the relevance and credibility of policy recommendations and of the extent of scientific–policy alignment, including potential intermediary roles (question 2.7). Finally, respondents were asked about the actions they would typically take after reading a policy brief (question 2.8), providing insight into its potential influence on policy processes.

Section 3 focused on preferences related to the design and presentation of policy briefs. It identified features that make briefs less appealing (question 3.1) and explored both positive and negative attributes across examples (question 3.2), as well as preferences for aesthetic and structural elements (question 3.3).

Section 4 adopted a broader, reflective perspective on the role of policy briefs. It explored respondents’ views on what constitutes a “good” versus “bad” brief and the features considered essential (questions 4.1 and 4.2), as well as distinctions between policy and research briefs (question 4.3). It also examined the circumstances under which policy briefs are most likely to inform decision-making (question 4.4) and how their usefulness compares with other forms of scientific communication (question 4.5).

### 2.2 Participant recruitment

Purposive sampling was used to recruit the interviewees from contacts of the research team through their work with Scottish Government and other UK administrations. Participants were eligible for inclusion if they were currently working in policymaking or policy advisory roles related to animal health and disease control, and if their role involved engagement with scientific evidence to inform decision-making. Veterinary epidemiology and animal disease control in the UK is a small, specialised field with extensive inter-agency working, and prior knowledge of the participants permitted targeted recruitment of information-rich individuals with extensive knowledge and experience of the topic. The established connections between the organisations of the interviewers and participants, and in some cases, existing working relationships between the two individuals, supported frank dialogue and co-production of knowledge during the interviews, although the interviewer and other authors responsible for data analysis remained aware of the potential issues around dual roles (11).

Six policymakers and eight scientists in policy advisory roles related to animal health participated in the study, resulting in 14 interviews. Six participants were male and eight were female. The length of time that participants had worked for their current organisation ranged from 11 months to 30 years, and most had experience of working in different policy areas within or outside of their current organisation. Most participants (*n* = 12) had scientific educational backgrounds, including seven with a veterinary degree. Nine had completed postgraduate degrees (PhD: *n* = 5, MSc and equivalent: *n* = 4). Only two participants had non-scientific educational backgrounds. The authors’ ongoing working relationships with Scottish Government resulted in many participants (*n* = 9) being recruited from Scottish Government, with the remaining five from other UK administrations. Seven of the Scottish Government participants had worked with EPIC directly and/or read EPIC policy briefs previously. Four out of five participants from other organisations had worked previously with EPIC and one had read EPIC policy briefs. Participants were approached via email and all of those contacted agreed to take part in the study.

### 2.3 Data collection

All interviews were conducted by one of the authors (MF), a female veterinarian and researcher holding a PhD, who was known personally to some of the interviewees through her role as an EPIC Knowledge Broker at the time of the interviews. All interviews took place in January and February 2022 and were conducted remotely via video call due to restrictions on travel and in-person meetings during the COVID-19 pandemic. Only the interviewee and interviewer were present on each call. Interviews were audio-recorded and then professionally transcribed by an external company. Interview transcripts were not returned to participants for correction. The interview recordings lasted between 31 and 78 min. Data saturation was achieved in accordance with the principle that the researchers were confident further data collection would not provide additional information (12).

### 2.4 Data analysis

All transcripts were coded by both KA and DP using the NVivo software. KA is a female veterinarian and social scientist (PhD) with experience in qualitative analysis of interview data relating to animal health. During her involvement in this research, she originally held the role of Knowledge Broker following MF, then moved into a leadership role within EPIC. DP is a male veterinary epidemiologist (PhD) who held the role of Knowledge Broker following KA. All of the authors have personal experience of preparing policy briefs on animal health topics for Scottish Government.

Thematic analysis was used to analyze the interview data. A method originating in qualitative psychology research, it provides a flexible and accessible approach for “identifying, analysing and reporting patterns (themes) within data” (13). The two major themes of i) the policy brief itself and ii) the policymaking process were determined in advance by the interview schedule design, as described above, and preliminary coding was a deductive process of allocating these major codes to the data. The sub-themes were developed inductively during the coding process to add depth and insight into the interviewees’ experiences to the two researcher-led major themes. In the context of the existing working relationship between the participants and researchers and the goal of co-production of knowledge through the interview discussions, this approach combined the *a priori* topics driven by the interview structure with a more reflexive approach to the interviewees’ responses.

## 3 Results

The interviews began by eliciting participants’ preferences for specific example templates. The results showing which templates were preferred are presented separately from the coding of the qualitative interview data, derived from conversations about the specifics of the presented examples as well as more general discussions of policy briefs as knowledge exchange tools.

### 3.1 Preferred template style

**Figure 3:**
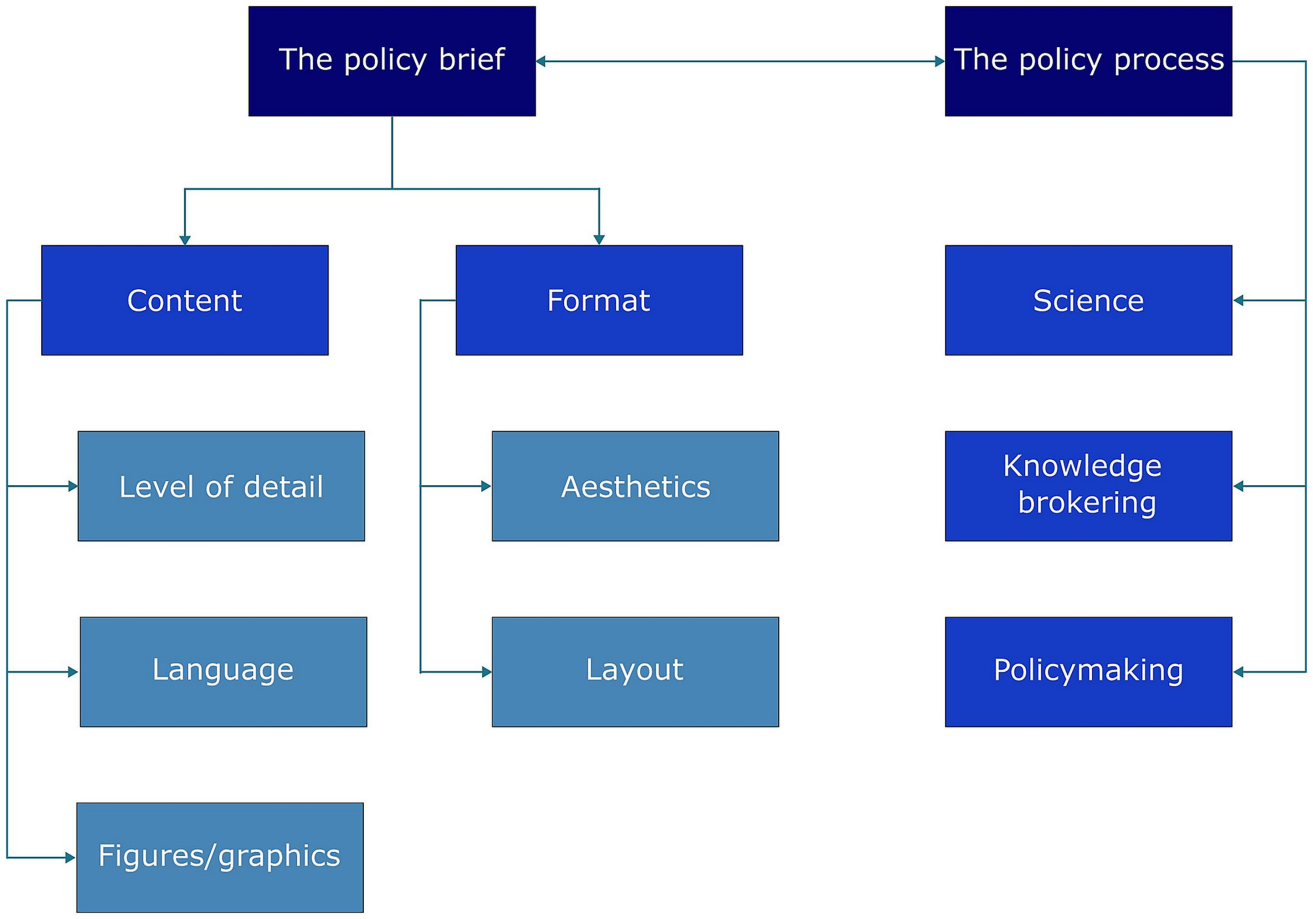
Coding tree used to analyze interviewees’ responses.

During the interviews, each participant was asked to select their favourite and least favourite examples from the four presented templates (Figure 2). The first template was chosen as the favourite by 1 interviewee and as the least favourite by 3. The second and third templates were each chosen as the favourite by 4 interviewees and as the least favourite by 2. The fourth template was most frequently selected as both the favourite (*n* = 5/14) and the least favourite (*n* = 7/14). While this is a very small sample and was not intended to be in any way representative of wider opinion, these results nonetheless demonstrate that the template preferences among the interviewees varied considerably. The reasons described for these preferences contributed to further exploration of decision-makers’ perceptions of policy brief documents and how these integrate with the policy process in animal health and disease control. An overview of the themes is presented in the coding tree in Figure 3.

### 3.2 The policy brief

Through the discussion of the example templates and further open questioning, participants’ views on the policy brief as a source of information were elicited. The sub-themes identified were: content (level of detail, language, figures and graphics) and format (aesthetics and layout).

#### 3.2.1 Content

The theme of “content” reflects the factual information covered within policy briefs and how this is conveyed to the intended audience, as distinct from the design of the brief covered in the “format” theme. In essence, this relates to the substance of the brief as opposed to the style, and is explored in greater depth through the three further subthemes.

##### 3.2.1.1 Level of detail

The level of detail required to report the scientific information covered by a policy brief was a recurring topic throughout the interviews. Highly detailed descriptions of the background to the research and the scientific methodology were not considered desirable (*n* = 12/14). However, a brief summary providing essential background information was considered necessary by most participants (*n* = 12/14) to inform a reader with limited experience of the topic. A summary of the methodology used, without providing excessive technical detail, was also required by interviewees (*n* = 9/14).

What information to include and exclude is a central challenge for a scientist writing a policy brief. Policy briefs must achieve a delicate balance between enough, but not too much, information for the intended audience. The inherent difficulty in achieving this balance was recognised by interviewees, along with the potential pitfalls if it is not achieved. The need for appropriate levels of detail to be made available was emphasised, to reflect the differing levels of detail required by multiple decision-makers who may view a policy brief at different times and across different roles.

*“The problem there is of course sometimes there isn’t a simple thing about it, you know? Sometimes things are inherently complex. And I suppose there’s a very difficult thing to actually simplify things when they are quite complicated. And there’s also the fact that if you oversimplify things it can look like a cover-up.”* (Interviewee 1)

*“If we simplify the message in the wrong way, it means that the minister won’t be able to really make the right decision.”* (Interviewee 10)

It was widely agreed (*n* = 10/14) that key messages summarising the main findings of the work should be identified and presented clearly so that they are immediately available to even a casual reader of the brief. The design approach to highlight key messages visually is also explored further within the “format” theme. Even within a single policy brief, which in itself condenses complex information into a more digestible form, the core messages within the brief must be summarised further and then supported by more detailed, but still accessible, evidence.

*“I would say not a huge amount of detail, maybe three main takeaway messages from the results, something like that.”* (Interviewee 14)

Some participants (*n* = 8/14) mentioned that these additional details should be made easily available if required or desired, but may not need to be presented within the brief itself to avoid an overwhelming level of information for the reader. This could instead be achieved by providing supplementary reports or appendices for reference.

*“I think the important thing is that the link is there to the detail if they want to go into that, and 95 per cent of the time they won’t want to.”* (Interviewee 13)

Another crucial aspect of effective policy briefs is communicating any uncertainties or reservations about the findings and recommendations presented. While most interviewees accepted that scientific evidence is never without limitations, they also spoke of the need for clear, unambiguous take-home messages from policy briefs to inform decision-making. A balance between communicating unavoidable uncertainty and providing robust information is needed. This discussion perhaps also illustrates the need for clarity in communicating uncertainty to convey whether more data or further analysis would provide greater certainty, or whether this is the inherent and unavoidable statistical uncertainty present in most outputs from quantitative research.

*“The assumptions are at the end, which no one ever reads, certainly not policymakers… they don’t like dealing with uncertainty… a policymaker doesn’t see his role as quality assuring the work.”* (Interviewee 13)

##### 3.2.1.2 Language

The language and vocabulary used in the policy briefs must be comprehensible to a reader without prior knowledge of the topic at hand. Most interviewees (*n* = 9/14) spoke of the need for simple language in briefs, using words that would be easily understood by a reader without specialist knowledge, and avoiding of scientific jargon. Minimising the use of abbreviations as far as possible, or at least ensuring that a clear explanation is included, was also recognised as good practice when writing policy briefs.

*“Good [policy briefs] are clearly written and they are concise and they don’t use too many scientific terms that non-scientists won’t understand.”* (Interviewee 3)

From the example templates, specific examples were identified. For instance, the word “stochastic” was cited by some interviewees (*n* = 4/14) as unsuitable for inclusion in a policy brief. While this is standard statistical terminology, the term is unlikely to be familiar to someone without statistical training, creating a barrier to comprehension. The word “standstill” (a legally defined period in which animal movements are not permitted in order to minimise disease transmission) was also picked up as another potentially unfamiliar term (*n* = 3/14).

*“If you are talking about a standstill, potentially it would be good to have somewhere an explanation of what standstill is, although I wouldn’t need it and nobody probably in my animal disease policy team would need that explanation—but our minister would.”* (Interviewee 10)

##### 3.2.1.3 Figures and graphics

The use of diagrams, charts and figures to communicate scientific evidence, in addition to the core text, was generally appreciated by the participants, provided that such figures were clear and easy to interpret (*n* = 10/14). In the examples presented, the use of maps to illustrate the implications of a potential policy change in a real-world context was received most positively. Visual summaries of the core information in the brief were welcomed, but participants emphasised the need to ensure that diagrams, charts and other approaches to data visualization added genuine value to the brief and could easily be understood.

*“I’m drawn to the graphics, I immediately want to see what the chart says because it’s a very strong visual indicator.”* (Interviewee 8)

*“So, the comprehensibility, the understandableness of the figures is really important. I mean, they shouldn’t just be pictures, they should be actually… working really hard to convey information.”* (Interviewee 4)

The connection between the text and graphics or figures was a recurring consideration for the participants. It was recognised that some individuals—particularly those with a scientific background—may find visual representations of data to be more useful (*n* = 7/14), while others will prefer a textual summary (*n* = 2/14), and that the inclusion of both options can be beneficial (*n* = 2/14).

*In a scientific paper I might leave it for somebody to take their own assumptions from those figures, but particularly in a policy brief, I would spell out a little bit in more detail what those figures are showing for them.* (Interviewee 14)

However, interviewees also mentioned that the graphs and figures included in the examples were too small to be read easily (*n* = 6/14). The challenge of balancing legibility with the need to fit figures into a limited space was acknowledged.

*“I like the ones with graphs and maps, but, on the other hand, they’re all too small, which…but then, that’s a juggle, isn’t it, keeping it to short documents.”* (Interviewee 3)

#### 3.2.2 Format

The format of the policy briefs (i.e., the visual design aspects of the documents, such as colour, illustrations, font, layout and the order in which information was presented) was explored through the interviews, centred on the example templates but diverging into wider discussion of what makes a good or a bad policy brief. The responses are broadly grouped into two subthemes: aesthetics and structure.

##### 3.2.2.1 Aesthetics

The visual appeal was identified by most participants as an important consideration for the preparation of good quality policy briefs (*n* = 11/14). Aspects such as the chosen colour scheme and font were perceived to substantially impact the attractiveness of the brief. However, the participants’ aesthetic preferences were highly diverse, and the feedback on the template examples presented in the interviews varied widely. The relevance of aesthetic appeal was described as important to ensure that the brief would catch the attention of busy policymakers, who may have limited time and attention to devote to a particular topic due to the need to juggle multiple, competing demands. There was also a perception among some participants (*n* = 4/14) that a visually appealing document would be easier to read.

*“If the font and colour of the background is very off-putting then it can be difficult to read and therefore difficult to take it in.”* (Interviewee 9)

*“I think that’s what your brain’s doing when you first see the document, you’re thinking, has this person thought about how I understand this document.”* (Interviewee 11)

The use of pictures for illustration, as used in examples 2, 3 and 4 (Figure 2) was generally positively received, but two participants clarified that any images used should be directly relevant to the topic of the brief. The species of animal should be appropriate to the topic of the brief, and even the breed of animal portrayed should be accurate to convey that the authors of the policy brief are familiar with the topic to inspire confidence in the reader.

*“If it’s about sheep in Scotland, then don’t use a picture that is of a breed that you’d only get in the south of England or something like that.”* (Interviewee 3)

There was some concern expressed that the use of illustrative images might detract from the formality or seriousness of the topic, but this was generally considered secondary to the benefits of enhancing the brief’s visual appeal and signposting the topic to a casual observer. Three participants felt that the presence of such images helped to remind them of the reason for their work, or that it was an immediate introduction of the subject of the brief before any further reading.

*“I like the pictures being in there and I don’t think that it takes away from the seriousness of the paper, if you know what I mean. I don’t think it trivialises the paper to have a picture of a lamb at the top of it.”* (Interviewee 2)

*“I think I would focus better on a document without pictures, but I wouldn’t remember it.”* (Interviewee 12)

The potential symbolism of the images used was also described by one participant:

*“I mean, the sheep climbing up a hill, so almost immediately you have an impression this is going to be an uphill struggle… or the findings might be a little bit woolly.”* (Interviewee 1)

Accessibility issues that may impact readers’ ability to engage with the documents, such as colour-blindness, visual limitations or learning disabilities, were mentioned by two participants. The existence of general guidelines to enhance accessibility of written documents was mentioned and it was suggested that adherence to these standards would be good practice when producing policy briefs (14).

##### 3.2.2.2 Structure

The order in which the various sections of the brief are presented was viewed as important for comprehensibility. The traditional flow of the scientific narrative, from background through methods to results and conclusions, was well received, but starting the brief with the key messages was generally felt to be the best way to ensure that these are instantly accessible to a busy reader (*n* = 11/14).

*“I think having the key message right at the top of the paper is the best.”* (Interviewee 2)

“So I like the ideas where things like the key message or the policy implications are at the top so you can see it and you see immediately is this something that is valuable to me now or is it something that I can leave later or for reference for the future? That’s valuable in terms of basically time management.” (Interviewee 1)

Some interviewees (*n* = 4/14) made a distinction between “key messages” as the main scientific findings and “policy implications” as the importance of these findings for a policy audience. It was felt that the policy implications should be presented as a conclusion based on the scientific evidence presented rather than as headlines to the document:

*“I think pulling out the key message and putting it at the top is much easier to read. I just don’t like the policy implications being at the top of it. I want to read about and go through the process before and think about the results and then read what EPIC are saying are the policy implications and then having a think about that myself.”* (Interviewee 9)

Opinion varied on the preferred way in which the information is laid out in the document. For example, some participants liked the text to be arranged into multiple columns (*n* = 8/14), while others favoured a single column (*n* = 4/14). Bullet points were, however, generally preferred to large blocks of text (*n* = 11/14).

*“I love reading, but the sight of a block of text, if it’s not a novel, can be really off-putting to me.”* (Interviewee 4)

The substantial knowledge from the communications sector on how to present information effectively was identified, suggesting that the approach to formatting and presenting policy briefs should also be evidence-based.

*“You know, I’m sure the chief vet would rather have a policy document laid out like this to read than something that was, you know, one single block of text, so I think those tricks work on everyone, in terms of the way the brain reads and takes in information, and then of course there’s a lot of science behind that already, isn’t there?”* (Interviewee 11)

The interviewees’ thoughts about the appropriate length of a policy brief again captured the challenging balance of providing enough information without overwhelming the audience, as also identified within the Content theme. In practical terms, one to two A4 pages in length was broadly agreed to be optimal (*n* = 10/14), although this could require Supplementary Material to be made available.

*“I think that [two pages] should be an absolute limit though. I don’t think, I don’t think you should go onto a third page, even though I realise it’s a struggle when you want to show a few pictures and things. I think it is worth keeping them small enough to fit everything on two sides.”* (Interviewee 3)

*“If you do a one pager then give me the annexes as well because I might want to… I’ll have experts as well that I might want to double check what’s been sent to me.”* (Interviewee 8)

### 3.3 The process

As well as the details of how scientific information should be presented and communicated through policy briefs, the interviews also explored the role of policy briefs in the knowledge exchange process at the science-policy interface. Knowledge brokering, where contact and exchange of ideas occurs between scientists and policymakers, often through an intermediary, is a key aspect of EPIC’s innovative model, as shown in Figure 1 in the Introduction. This established model for science-policy working, including specialist knowledge brokers, was reflected in the discussion. The views of policymakers on the role of the policy brief at each stage of the process are explored in the context of the three main activities identified: policymaking, knowledge brokering, and science.

Communication of scientific evidence for policy making can be viewed simplistically as linear and unidirectional, from the generation of scientific evidence by academic researchers through to the application of that evidence by policymakers to develop and implement evidence-based animal health policy decisions. However, as demonstrated by the interview findings, this process should ideally take the form of a dialogue between scientists and policymakers, with an ongoing conversation and exchange of ideas as the best way to share evidence and achieve policy aims.

#### 3.3.1 Policymaking

The demands on policymakers were emphasised by the interviewees, citing lack of time, large volumes of information to absorb and assimilate, and the pressure to make effective decisions quickly, particularly in the event of an active disease outbreak (*n* = 6/14). Within this context, the need for policy briefs to be concise, accessible sources of actionable information was clear.

*“Sometimes policymakers don’t have time to read a lot. Now that all depends on the luck of when a paper is issued, so it emerges in the middle of an epidemic of a disease then, as I found out practically, the volume of emails that pour onto your computer is huge. There’s not just me, other people are getting a bit confused by it and stressed out, because you feel you should know everything that’s coming in.”* (Interviewee 1)

*“The policymaker isn’t necessarily interested in the things that are good to know, they want to focus on what needs to be done.”* (Interviewee 10)

A policy brief addressing a question determined by policymakers attracted more interest from the interviewees than one based on a question originating with scientists. Some participants (*n* = 4/14) expressed their belief that a policy brief should be used only to present evidence which informs pre-agreed policy decisions directly, rather than purely scientific outputs where the policy application is not clearly defined. Policy-focussed research will often arise from external events, such as a disease outbreak or other specific reason that the issue is being addressed. The presence of an imminent threat is also most likely to result in rapid action based on the evidence presented in a policy brief (*n* = 4/14).

*“Usually the drivers to change policy are from industry or from ministers or from some external factor, you know, where the situation just changes.”* (Interviewee 2)

*“It does need to relate back on, why do government care about this?”* (Interviewee 9)

*“If the policy brief was to highlight any immediate unacceptable risk to human or animal health, I think that would be a major prompt for action.”* (Interviewee 2)

Policy briefs were viewed by the participants as a snapshot of evidence at a point in time, primarily to raise policymakers’ awareness and assist with prioritisation of an issue or risk requiring attention. Ultimately, policy decisions utilise a wide range of information from multiple sources, with a policy brief representing just one source of evidence. In some cases, even if the science points to a certain course of action, other considerations may result in a different decision being made, including no change to policy.

*“From a policymaker’s point of view, the starting point for them making policy is the scientific basis for it. But then they have a whole lot of other economic and social political and legal factors that have to be used to derive the final policy.”* (Interviewee 13)

The core purpose of a policy brief, as identified by interviewees (*n* = 8/14), was to inform policy decisions—either by evidencing whether a policy change is required, or by indicating what change should occur to achieve a desired result. The evidence presented in policy briefs (and via other channels) fulfils a dual role in policymaking. Firstly, it identifies whether change is required and informs decision-making from an early stage of the policy process. Secondly, the evidence presented can be used to support and defend the decisions made if required, usually in response to queries from members of the public, to increase public confidence and prevent reputational damage to government.

*“It’s subject to freedom of information requests, so someone might want to see the research that underpins a certain policy, so it needs to be fully auditable and transparent.”* (Interviewee 8)

Trust in the source of the evidence presented is vital for effective translation to policy. Some interviewees (*n* = 3/14) implied that the methods and interpretation from trusted sources may be given greater weight in decision-making and receive less scrutiny, than if evidence were presented from an unknown or untested source. Professional relationships between policymakers and known scientists, often established through working together for an extended period of time, strengthened this trust further. This was sometimes contrasted with the lack of trust in decision-making from the general public.

*“In the policy role… there’s the trust in the people who were the authors and did the research for the reports and there’s the distrust by members of the public and the people out there. What you find is you take an action in policy and then members of the public come back and say but where’s the evidence? Why are you doing this?”* (Interviewee 1)

#### 3.3.2 Knowledge brokering

Knowledge brokering refers here to activities that promote knowledge exchange between scientists and policymakers, and is a core component of EPIC’s established approach to science-policy communication. EPIC Knowledge Brokers are interdisciplinary scientists who conduct scientific work in response to Scottish Government requests and facilitate the communication of scientific outputs between other EPIC scientists and policymakers. As such, EPIC Knowledge Brokers maintain a strong understanding of current policies and the needs of policymakers while remaining actively engaged in scientific work.

The role of an intermediate person to act as a broker when preparing policy briefs and other scientific outputs for a policy audience was discussed in the interviews. This sub-theme covers knowledge brokering both as a distinct role at the science-policy interface, and as direct interactions between scientists and policymakers. Opinion on the utility of individuals acting as dedicated knowledge brokers was divided. Some interviewees (*n* = 7/14) recognised the importance and value of having an intermediary to facilitate communication between the authors of a brief and policymakers, while others (*n* = 2/14) expressed scepticism about the knowledge broker role due to concerns that it might compromise the independence of other scientists.

*“Translating science into policy is not everybody’s strength and where there’s, you know, people that are fantastic researchers but don’t quite have that skill in translating that into policy and making it clear.”* (Interviewee 9)

*“I don’t think you need a named individual. I think you just need close liaison between the people who are assessing the policy question and the scientists who are answering it.”* (Interviewee 6)

Direct interaction between policymakers and scientists was also explored, and policymakers identified the need for ongoing engagement for effective communication (*n* = 5/14). The policy brief is one tool that can be used within knowledge brokering, but it is most useful as one component of an ongoing conversation between scientists and policymakers, with or without an intermediary. The challenges for some scientists of communicating their work in accessible terms were also identified.

*“It really does need to be presented and discussed. There does need to be that interaction between scientists and policy and it can’t purely be… It can never purely be, here’s a bit of paper with your answer on it.”* (Interviewee 13)

*“[Scientist X] is a good example of someone who is, [they’re] so smart that it’s really hard for [them] to dial it right down to clarify it.”* (Interviewee 4)

#### 3.3.3 Science

Scientists generally produce a policy brief close to the completion of their research, when results are available and there is an opportunity for reflection on the potential impact of the findings. Policy briefs are likely to be produced alongside other outputs such as reports, peer-reviewed papers or presentations. These other scientific outputs were recognised by participants as an important aspect of the underlying evidence generation as part of quality assurance (*n* = 7/14), but were not viewed as directly relevant to policymaking by most participants (*n* = 10/14).

*“And then the publications, I very rarely read publications these days because I don’t have the time, but they’re important to the researchers, as you know, for a number of reasons, really, partly that you have to do it. We expect it to be done, it’s an audit. It’s peer review, it’s the world and us knowing that the work is of a standard that’s publishable so we don’t have to have that knowledge or time input.”* (Interviewee 6)

*“Policy briefs have to be…how do I put this? The policy brief should really be a bit more attuned to impact on real life, you know what I mean. Like a research paper can be quite independent of real-world impacts, whereas a policy brief should be focussed on how this could change things in the near to medium future.”* (Interviewee 2)

Despite this, the importance of scientific independence to ensure that the evidence base presented in a policy brief is robust and unbiased was emphasised in the interviews. Scientists’ integrity and impartiality were highly valued. It was felt that scientists must have a good understanding of current policy to write effective policy briefs, and that engagement with policymakers throughout the research process benefits the final output (*n* = 12/14). A thorough understanding of the relevant livestock sectors and how they operate was also considered to be important to provide context to a policy brief. However, scientists were not expected to have a full grasp of the range of issues that policymakers must consider, or of the intricacies of the policymaking process.

*“You start from the science, you start from the ideal, this is ideally what we should do and then it can be watered down by other people… I don’t think it’s [scientists’] role to take into account other things like finances or logistics or anything else, because I think there needs to be an ambassador for science, the people who do it.”* (Interviewee 14)

*“The more information that scientists have of policy and vice versa, then the more intelligent the conversation is.”* (Interviewee 11)

## 4 Discussion

The findings from this study provide insight into the requirements of policymakers as end-users of policy briefs in the field of animal disease control. The desired outcomes were identified, both in terms of the content and formatting of the brief itself, to meet the needs of the intended audience, and in terms of the role of policy briefs as a tool for communication at the science-policy interface.

Based on the findings from this research, EPIC Scotland has made changes to its internal processes for policy brief writing, including a consistent process for knowledge brokering support whereby all outputs receive an internal review, and the creation of an updated and consistent template for policy briefs and other outputs, developed by a communications specialist. There is also now a recognised distinction between Research Briefs, which provide updates on scientific evidence which may be of general interest, and Policy Briefs, which address a specific policy question. Preliminary feedback from our policy audience has been positive and further evaluation of the current process will continue. As an internationally recognised model of excellence in working at the science-policy interface for animal disease control, these practical changes to EPIC’s policy briefs may catalyse similar changes in other settings.

### 4.1 The policy brief—achieving balance

The process of creating an effective policy brief requires the author(s) to achieve a delicate balance across every aspect of content and formatting. In terms of the content of the brief, the level of detail included must provide enough information to be useful without overwhelming the reader. The language used should be simple, accessible and engaging, while also maintaining the appropriate formal tone for communication with policymakers. Challenges in assimilating certain types of information were identified from the interview data, particularly concerning the communication of uncertainty in scientific research. Some participants, while acknowledging the importance of assumptions and limitations in policy briefs, admitted finding it challenging to interpret the real-world implications of the uncertainty generated by these factors and expressed a preference for clear and unequivocal guidance on how to act based on scientific evidence. This may occasionally conflict with the scientific ethical principles of honesty and transparency, which stipulate that scientists should report uncertainties as openly as possible (15). However, some scientists argue that communicating highly uncertain estimates to policymakers is more likely to result in inaction (16). In this context, too, a balance must be achieved between presenting uncertainty as factually as possible, while also providing practical recommendations for implementation.

The concept of “balance” also applies to formatting and design to create a document that is visually appealing and easy to read for a busy policymaker, while remaining professional and informative. In contemporary society, we are constantly overwhelmed by information in both our personal and professional lives (17). As a result, our ability to focus is continually challenged, increasing the risk of overlooking relevant information that fails to capture our attention. For this reason, policy briefs should be thoughtfully curated from the perspectives of both content and visual presentation. While the presence of visual embellishments, such as pictures and colours, may not be everyone’s preference, this study found that they were generally appreciated, as supported by previous research (6). Where pictures are used, the interviewees emphasised that such images must be correct in every detail, down to the breed of livestock represented, suggesting that pre-testing with an appropriate audience may be beneficial. Enhancing the visual impact of a policy brief was identified as an important way to capture participants’ attention, with one interviewee noting that visually curated briefs are also more likely to be remembered. Increased memorability has also been reported as a benefit of visual embellishments in other studies (18). However, caution should be exercised when designing a policy brief to avoid having decorative elements divert the reader’s attention from its content.

In this study, interviewees expressed varying opinions on how to achieve a visually appealing brief. We found that there is no single, optimal policy brief template that will satisfy the individual preferences of all readers. While it may be unrealistic to expect a template that meets everyone’s preferences, we were nonetheless able to identify elements that were universally considered to make a brief visually unappealing. These included cluttered text, the use of small font sizes, and decorative images that are inappropriate or unrelated to the subject matter discussed.

The policymakers interviewed recognised that a document that strikes the right balance across both information and design can be challenging for scientists accustomed to presenting their work to a specialised peer audience. The role of a knowledge broker in supporting this process and achieving the desired balance was understood and appreciated by some, but not all, interviewees. It must be considered that not all interviewees were familiar with the established role of the Knowledge Broker within EPIC. There may be a need to ensure that the value of this role is communicated clearly to policy audiences, and to manage the expectation among some recipients that scientists should be able to produce policy briefs independently that achieve the balance desired by policymakers.

### 4.2 Trust—situating the brief in the policy process

The policy brief document is a commonly used tool for knowledge exchange at the science-policy interface, where “trust” has been identified in previous research as a central component (6, 19), as well as in this study’s findings. A well-crafted policy brief that achieves the balance in content and format to meet the requirements of the audience serves to build and enhance this trust. Policymakers’ trust in the brief, in the underlying science, and in the scientists who have generated the relevant evidence and produced the brief is essential. Underlying this trust is the willingness and need for policymakers to accept the content of a policy brief without having to challenge its robustness. This process is influenced by two main constraints faced by policymakers: a lack of time and expertise. The best way to build trust, as reported by the interviewees in this study, is for scientists and policymakers to regularly engage in conversations focused on the progress of policy-relevant scientific work and the needs of policymakers. Knowledge brokering activities, such as those implemented by EPIC, can promote this trust (20). Supporting public trust in government is also at the heart of effective policymaking, and accessible documents such as policy briefs provide policymakers with assurance that their decisions are based on robust evidence, as well as providing an auditable and shareable resource in the event of future questioning of decisions, either within government or from stakeholders. Through consistent and meaningful engagement, building and maintaining trust can benefit both policymakers, who gain confidence in the quality of the work presented and are more likely to act on it, and scientists, whose research is more likely to have a positive impact on society.

### 4.3 Recommendations for improving communication with decision-makers using policy briefs

**Figure 4:**
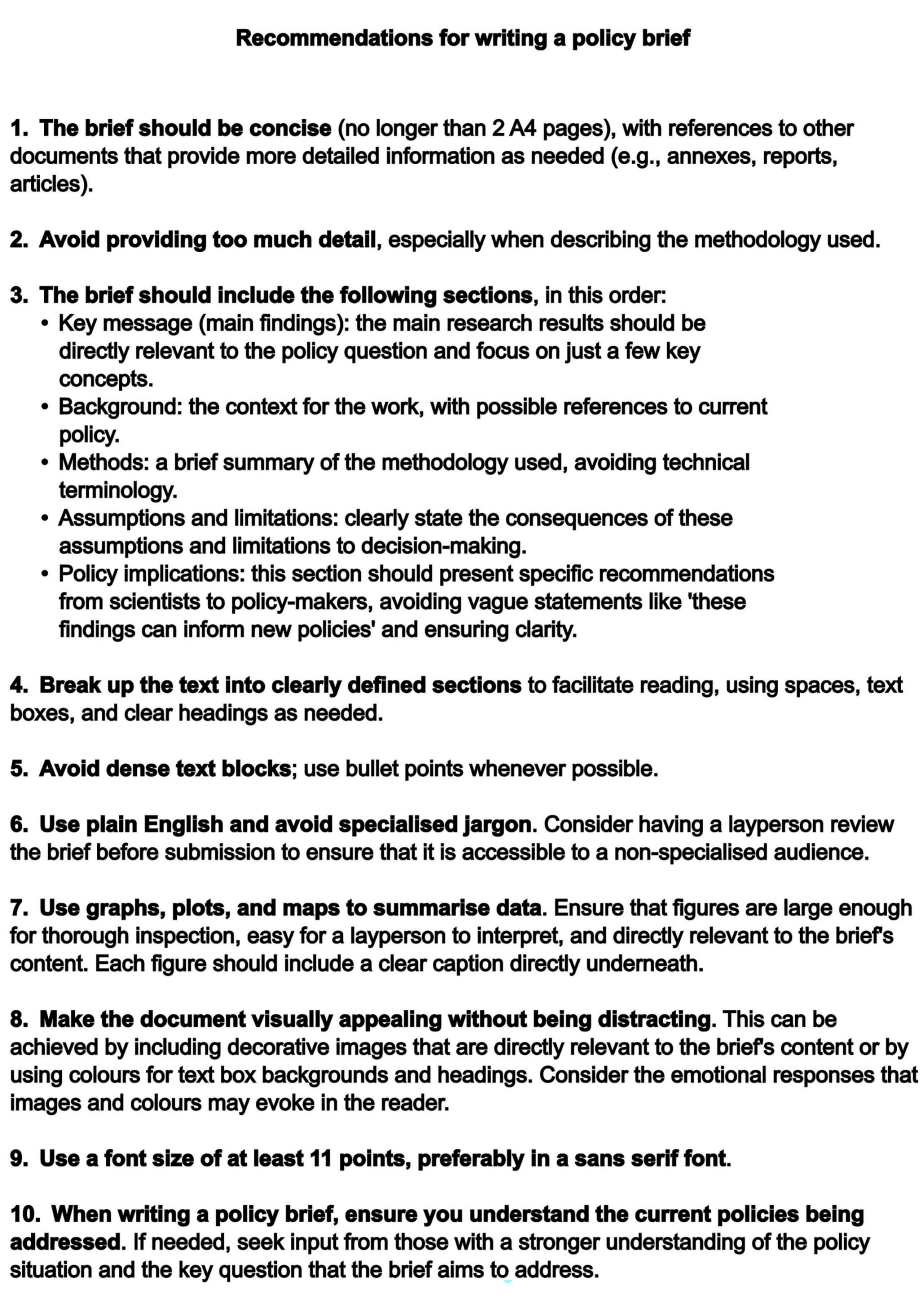
Recommendations for writing a policy brief.

Building on the findings presented above, a set of evidence-based recommendations for improving communication with decision-makers through policy briefs was developed (Figure 4). These recommendations synthesise the key principles identified across the interviews, with particular emphasis on achieving balance in content and design and fostering trust at the science–policy interface.

A central implication of the findings is the need to prioritise conciseness and accessibility. Policy briefs should focus on communicating essential information clearly and efficiently, recognising the time constraints under which policymakers operate. This includes limiting unnecessary technical detail while ensuring that sufficient information is available, either within the brief or through Supplementary Materials, to support informed decision-making.

Clarity of structure is equally important. The consistent emphasis on prominent key messages reinforces the need for policy briefs to provide an immediate and accessible synthesis of the main findings and their implications. Supporting sections, including background, assumptions and limitations, and policy recommendations, should be presented in a way that facilitates rapid understanding while maintaining transparency, particularly in relation to uncertainty.

The findings also highlight the importance of presentation as a component of effective communication. Readable layouts, a clear visual hierarchy, and the use of concise formatting choices, such as bullet points, can support understanding and engagement. Visual elements, including figures and design features, should be used judiciously to enhance communication and memorability without detracting from the core message.

Language plays a critical role in bridging the gap between scientific evidence and policy application. The use of plain English and the avoidance of specialised terminology are essential to ensure that policy briefs are accessible to non-expert audiences, while still conveying the necessary level of precision.

Finally, the importance of situating policy briefs within their policy context underscores the need for alignment between scientific outputs and policy priorities. This includes demonstrating an understanding of current policy frameworks and clearly articulating how the evidence presented can inform decision-making. Strengthening collaboration between scientists and policymakers, including through knowledge brokering roles, may further enhance the relevance and impact of policy briefs.

### 4.4 Limitations

While the sample size for this study may be considered a limitation, the number of interviews conducted is consistent with in-depth qualitative research using thematic analysis. Previous research suggests that data saturation can be achieved with a minimum of nine interviews, particularly for more homogeneous study populations such as the one examined here (21, 22). Although the sample size limits the generalisability of the findings, this is not the primary objective of the study. Instead, the depth of the information presented should allow readers to assess whether the findings are transferable to a different context.

The data collection was conducted entirely within the context of animal health and disease control policy in the United Kingdom, with most respondents drawn from the Scottish Government. In Scotland, policymaking is supported by long-established Centres of Expertise, which bring together scientific consortia to inform decision-making. EPIC, as one of these Centres of Expertise, has operated for over a decade, fostering close working relationships between scientists and policymakers through ongoing collaboration and regular exchange of evidence (10). This context is likely to have influenced both participants’ expectations and the ways in which policy briefs are used and interpreted in practice. As a result, the findings may be less applicable to settings where trusted relationships are not established.

At the same time, some aspects of the findings are likely to be relevant beyond this specific context. Similar challenges around communicating complex evidence to non-specialist audiences are likely to arise in other areas of policy that rely on scientific input, such as public health, environmental management, or food safety. In these contexts, the emphasis identified here on clarity, structure, and explicit links to policy decisions may be transferable, although their implementation will depend on local institutional arrangements and ways of working.

The findings must be interpreted in the context of the professional roles of the researchers and their existing close links to both the interviewees and the work explored through the interviews. Existing connections may elicit greater openness from participants, or conversely could result in less frank discussion than with an entirely unknown researcher. As the participants appeared willing to challenge current practice, even when this was a core aspect of the interviewer’s role at the time of the interviews, this implies a level of comfort with disclosing information truthfully. The tacit knowledge of the topic under discussion for both interviewer and interviewees may facilitate the ease of the conversation, but could also result in unspoken agreement on subjects that would have required more explicit discussion with a more independent researcher, potentially limiting the richness of the data. The separation of data collection and analysis between researchers will, to some extent, limit the analysis due to loss of non-verbal communication, particularly as the audio recordings were not available and the analysis was conducted using the transcripts only, but may support a more impartial understanding of the data. The double coding by two researchers sought to mitigate this and enhance the reliability of the results presented.

## 5 Conclusion

The findings from this study provide a unique insight into the needs and preferences of the intended audience for policy briefs in the field of animal disease control. These findings are likely to be of practical relevance to scientists working in this field who are involved in preparing outputs for policy. The process of writing a good policy brief is a constant balancing act to provide comprehensible and complete information. Our research concludes that there is no single preferred format that should be adopted for all policy briefs. As personal preference varies so widely among the policy audience, the main goal in designing a template for a brief should be to ensure that it complies with general guidance around accessibility (e.g., colour blindness or learning disabilities). Policy briefs are one of a range of approaches used to support science-policy communication, including both written outputs such as reports and verbal communication such as presentations, and are at their most useful when embedded in ongoing discussion of the topic of interest within the context of an established, trusting relationship between scientists and policymakers.

## Data Availability

The raw data supporting the conclusions of this article will be made available by the authors, without undue reservation.
