# Supplementary material for: What policymakers want: policy briefs as tools to inform animal disease control strategies
Source: Front Vet Sci. 2026 May 21;13:1813832. doi: 10.3389/fvets.2026.1813832 (PMC13235431; doi:10.3389/fvets.2026.1813832)
Supplement: Supplementary file 1 [file Table_1.DOCX]

Supplementary Material

# Full interview schedule

| **Section 1 – Background information** |
| --- |
| 1.1 Which organisation do you belong to?  1.2. What is your current role in the organisation?  1.3. How long have you worked in this organisation?  1.4. How long have you worked in your current role?  1.5. In what other policy areas have you previously worked?  1.6. Have you ever worked in collaboration with EPIC scientists?  1.7 Have you ever read an EPIC policy brief – if yes, could you please estimate how many?  1.8. What is your highest level of education, if you do not mind sharing?  (If university degree) What was your degree subject? |
| **Section 2 – Contents of example briefs** |
| 2.1. Why did you select this brief?  2.2. a. Can you easily identify take home messages of this brief by reading once? if yes, what are   they? If not, why that is the case?  b. What do you think is the most effective way to deliver key messages in the form of policy   briefs?  2.3. a. Are the background and objectives of study clear to you? And could you explain why?  b. What degree of detail in background and objectives would you like to see in the policy  brief?  2.4. a. Are the results presented in a way that you can understand how they relate to the policy  question, or do you need further explanation or other evidence to do this?  b. What degree of detail in the results would you like to see in the policy brief?  c. Do you have a preference about how the results are presented (e.g.,   bullet points vs narrative styles)? Please provide some examples.  2.5. a. What degree of detail in the methodology would you like to see in the policy brief?  b. How important is it for you to understand the methodology behind the key messages?  2.6. a. Are the assumptions and limitations of the work clearly explained (alternatively, can you   identify assumptions and limitations of the work from reading this policy brief)?  b. How do you take into account assumptions and limitations of the work when developing   evidence-based policies, and why?  c. Could you share your views on the most effective way to communicate assumptions and  limitations of the work?  2.7. a. To what extent do you agree with the policy implications and recommendations made by  the authors?  b. Could you elaborate why that is the case?   c. How well do you think scientists should understand current policies when they write a policy  brief?  d. Please suggest what you think is the best approach for scientists to write a policy brief that   is relevant to current policy issues – e.g., using an intermediate person like a policy analyst  or interpreter  2.8. Once you read a policy brief, what actions would you usually take within your policy team? |
| **Section 3 – Impression of example briefs** |
| 3.1. Which brief is least appealing to read? – could you briefly explain why (more detailed  questions will follow)?  3.2. Could you describe what you like/dislike about each of the briefs?   Do you normally print out policy briefs or read on the screen?  3.3. Could you share your views on/preference of font, font size, layout, colour, length, images,  and the order of presentation? |
| **Section 4 – general impression on policy briefs** |
| 4.1. In your opinion, what makes some policy briefs “good” and others “bad”?  4.2. What do you think is the most important thing to capture in a policy brief? (prompt: what is  the defining element that makes you consider a document, a “policy brief” – e.g., is it the  inclusion of policy implications/recommendations related to the work, or does there need to  be some evidence of greater understanding of current policies and an integration of this work  into new approaches etc.?)  4.3. What differences do you see between a policy and a research brief? (prompt: would you call   this a policy brief or research brief?)  4.4. In what circumstances are you most likely to act on the basis of information provided in a  policy brief (prompt: or least likely to act on that information?)?  4.5. a. There are other ways for scientists to communicate with policy makers (e.g., executive   summary, full technical report, peer-reviewed research article, email summary, or face-to-  face presentation). Under what circumstances do you think policy briefs are a more effective  way than these other approaches, for scientists to communicate their work with policy   makers?  b. Could you explain why? |
